# Correction Notice

**DOI:** 10.1080/07853890.2025.2588001

**Published:** 2025-11-16

**Article title:** Optimizing recovery in orthognathic surgery: the cohort study of impact of rocuronium–sugammadex on extubation and perioperative outcomes

**Authors:** Shao-Chun Wu, Tsung-Yu Wang, Amina M. Illias, Yung-Fong Tsai, Yi-Ping Wang, Ying-An Chen and Chun-Yu Wu

**Journal:** *Annals of Medicine*

**Bibliometrics:** Volume 57, Number 01, 2536201

**DOI:** https://doi.org/10.1080/07853890.2025.2536201

When the above article published online, it contained incorrect numerical values in the Abstract, Results text and in Table 1, Table 2 and Table 4. These have now been corrected and the article has been republished with the accurate information.

Abstract – Correction:

Incorrect: hospital stays (3 [3–5] vs. 4 [4–5] days, p = 0.003). They also exhibited lower inhalational anesthetic consumption (p = 0.002)

Correct: hospital stays (4 [3–5] vs. 4 [4–5] days, p = 0.003). They also exhibited lower inhalational anesthetic consumption (p = 0.044)

Results – Correction:

Incorrect: anesthetic (sevoflurane) consumption [12.96 (10.83–15.42) vs.10.63 (8.59–13.94) mL/h; p = 0.002]

Correct: anesthetic (sevoflurane) consumption [13.52 (11.12–17.04) vs. 12.24 (8.96–16.38) mL/h; p = 0.044]

Table 1 – Correction:

ASA physical class I p-value updated from 0.699 to 0.671

Table 2 – Correction:

PONV values updated to: 94 (26%), 71 (25%) and 23 (29.9%)

Table 4 – Correction:

Medical cost (USD) column has been removed.

